# Profound postinduction hypotension precipitated by immune checkpoint inhibitors: a case report

**DOI:** 10.1186/s13256-024-04375-9

**Published:** 2024-03-09

**Authors:** Lu Che, Afang Zhu, Wen Chen, Chunhua Yu

**Affiliations:** Department of Anesthesiology, Peking Union Medical College Hospital, Beijing, 100730 China

**Keywords:** Immune-related adverse event, Intraoperative hypotension, Immune checkpoint inhibitors, Perioperative hypothyroidism

## Abstract

**Background:**

With the increasing use of immune checkpoint inhibitors (ICIs) in cancer therapy, perioperative healthcare professionals need to be vigilant about potential immune-related adverse events (irAEs). We report a case of severe postinduction hypotension in a patient undergoing laparotomy due to suspected intraabdominal bleeding from gastric cancer and Krukenberg tumors, caused by unrecognized hypothyroidism precipitated by ICIs.

**Case presentation:**

A 65-year-old Chinese female with a history of gastric adenocarcinoma and Krukenberg tumors, previously treated with nivolumab, presented to the emergency room with abdominal pain and hypotension. Despite ruling out other causes, including hypovolemia and anaphylaxis, her hypotension persisted. The patient was found to have severe hypothyroidism, likely an irAE from the use of nivolumab. Thyroxine replacement therapy resolved the hypotension, and the patient recovered uneventfully after surgery.

**Conclusions:**

This case underscores the importance of considering irAEs, such as hypothyroidism, in patients treated with ICIs. Perioperative healthcare providers must remain vigilant for potential complications and promptly recognize and manage irAEs to optimize patient outcomes.

## Background

While immune checkpoint inhibitor (ICI) therapy has improved patient outcomes, it has also led to an increase in unique immune-related adverse events (irAEs) [1, 2]. Endocrinopathies caused by ICIs have been reported in up to 5% of patients, with hypothyroidism occurring in up to 8% of patients [3]. Anesthesia in the presence of hypothyroidism carries an increased risk of morbidity and potential mortality [4, 5]. It is crucial to stay updated on the field of immunotherapy and its associated toxicities, which can complicate perioperative management [6, 7]. Herein, we report a case of severe postinduction hypotension in a female patient scheduled for laparotomy due to suspected intraabdominal bleeding due to gastric cancer and Krukenberg tumors. Her hypotension was later found to be caused by unrecognized hypothyroidism due to immune-related adverse events (irAEs) related to nivolumab.

## Case presentation

A 65-year-old Chinese female presented to the emergency room (ER) with abdominal pain and one episode of syncope due to hypotension (70/60 mmHg).

Upon physical examination for the current emergency room visit, a palpable mass was identified in the lower abdominal and pelvic region, accompanied by tenderness and rebound pain. Neurological exam is insignificant with E3V4M5. The patient’s vital signs were stable, with blood pressure (BP) of 90/60 mmHg, oxygen saturation (SPO2) of 91% on room air, heart rate (HR) of 64 bpm with normal electrocardiograph, and arterial blood gas (ABG) analysis showing a lactate (Lac) level of 3.5 mmol/L.

Laboratory results revealed a platelet level of 93 × 109/L, hemoglobin (Hb) of 101 g/L, hematocrit (Hct) of 28.7% and creatine kinase (CK) level of 1928 U/L with other liver and renal function tests, urinalysis and complete blood count tests within normal ranges.

**Fig. 1:**
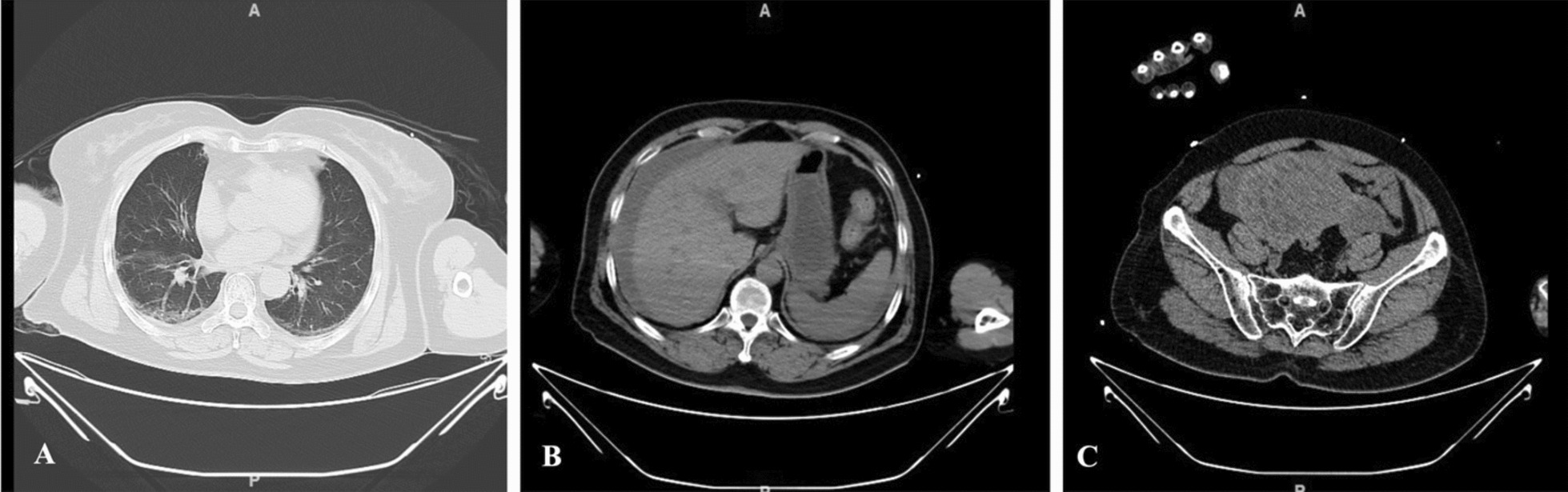
Preoperative CT scan. **A** Pulmonary CT scan showing subpleural arc-shaped opacities in both lungs; Small amounts of fluid in both pleural cavities; Small amounts of fluid in the pericardial sac. **B** Abdominal CT scan showing fluid accumulation around the liver and spleen. **C** Pelvic CT scan showing bilateral solid masses in the adnexal regions, possibly with associated bleeding; Small amounts of fluid in the pelvic cavity

An abdominal/pelvic computed tomography (CT) scan suggested possible bleeding in the uterine and appendix regions (Fig. 1). The patient's past medical history was significant for diagnosis of poorly differentiated adenocarcinoma in the gastric antrum 2 years ago, with initial tumor staging of cT4aN + . Following 3 cycles of FOLFOX treatment, Stable Disease (SD) was observed. Upon follow up newly identified seeding metastasis in the bilateral adnexal area led to a diagnosis of Progressive Disease (PD) with Krukenberg tumors. Then patient underwent 6 cycles of Nivolumab + XELOX with SD and tumor shrinkage achieved. Three months before the recent emergency visit, the patient had been on maintenance therapy with nivolumab + Capecitabine. During the treatment patients experienced iron-deficient anemia and one episode of upper gastrointestinal bleeding which completely resolved. There was no prior history of surgical procedures.

Peritoneocentesis performed in the ER revealed unclotted blood, indicating the need for emergency laparotomy. The patient's vital signs remained stable during her stay in the emergency room, with systolic BP ranging from 110 to 100 mmHg and HR ranging from 60 to 70 bpm. Her elevated CK was considered to be dermatomyositis caused by nivolumab, and methylprednisolone 40 mg iv every 24 h was given. Upon entering the operating room (OR), the patient was oriented, alert, and conscious (Glasgow Coma Scale: E4V5M6), with vitals similar to those in the ER and SPO2 at room air ranging from 85 to 88%. ABG analysis showed a pH of 7.43, Hct of 27.8%, Hb of 89 g/L, partial pressure of oxygen (PO2) of 72.9 mmHg, partial pressure of carbon dioxide (PCO2) of 32.6 mmHg, and lactate (Lac) of 4.7 mmol/L. Invasive arterial blood pressure monitoring was initiated, and point-of-care ultrasonography (POCUS) was performed.

The transthoracic echocardiogram (TTE) showed normal left ventricular contractility with no obvious valvular abnormalities or RWMA. The inferior vena cava (IVC) was not collapsed, and respiratory variability was not apparent. Abdominal ultrasound showed her bilateral diaphragm elevated to around the T4 level, likely due to the abdominal mass and possible bleeding. Fluid was observed in both the left and right abdominal cavities. Pulmonary ultrasound revealed B-lines in the lower lung regions, possibly caused by the elevated diaphragm due to the abdominal mass and potential intraperitoneal bleeding.

During the POCUS exam, the patient remained calm and cooperative, showing no signs of distress. Due to elevated intraperitoneal pressure, rapid sequence induction was performed to minimize the risk of aspiration. Propofol Targeted control infusion of 3 µg/mL, rocuronium 50 µg, and fentanyl 50 µg were used for anesthesia induction, and the Sellick maneuver was performed. Successful intubation was quickly achieved, but the patient's blood pressure started to drop. Boluses of 6 mg ephedrine and 100 µg phenylephrine were administered. The patient was quickly placed in the Trendelenburg position to compensate for vasodilation and facilitate central venous catheter placement. Despite repeated doses of ephedrine (36mg in total) and phenylephrine (1mg in total), the patient remained hypotensive at approximately 60/40 mmHg and unresponsive to fluid resuscitation(rapid infusion of 500ml lactate ringer’s solution). A bolus of 20 µg of epinephrine was administered together with epinephrine infusion and norepinephrine infusion, gradually improving the patient's BP. Throughout the hypotensive period, which lasted approximately 15 min, the patient's heart rate remained below 80 bpm. After establishing a central line and initiating a continuous infusion of epinephrine and norepinephrine, the hypotension was slowly corrected, and the patient’s invasive BP stabilized at 130/70 mmHg with an epinephrine infusion rate of 0.15 µg/kg/min and a norepinephrine infusion rate of 0.1 µg/kg/min. Surgery revealed hemorrhagic infarction of the Krukenberg tumor due to torsion of the pedicle as the source of intra-abdominal bleeding. The patient received 4 units of packed red blood cells. ABG analysis at the end of the surgery showed an Hb level of 9 g/L and lactate of 8.2 mmol/L, with an Hct of 27.6%.

The surgery proceeded uneventfully and lasted approximately 90 min. Despite the absence of continuous surgical bleeding, vasopressor support could not be downscaled. The patient was transferred to the intensive care unit (ICU) with continuous vasopressor infusion. Bedside ultrasonography was repeated to investigate the cause of severe hypotension after the induction agent. The patient's ventricular contractility was normal, without other apparent signs of valvular abnormalities or RWMA. The possibility of an anaphylactic reaction to induction agents was considered. However, the patient’s absence of signs and symptoms of anaphylaxis, including cutaneous and respiratory manifestations, led us to consider another possible cause: an immune-related adverse event (irAE). Considering the patient's history of taking nivolumab, a PD-1 inhibitor, together with the patient's unelevated HR during intro-abdominal bleeding and profound hypotension after induction, thyroid dysfunction was suspected. Thyroid hormone levels were ordered together with cardiac biomarkers to detect cardiac injury. The results on the second day revealed severe hypothyroidism, while troponins and NT-pro BNP remained unelevated (Table 1). An endocrinology consult was ordered, and thyroxine was promptly administered. The patient was free of vasopressors and successfully weaned off ventilation after 14 h in the ICU and transferred to the ward on the 2nd postoperative day, with an uneventful recovery. Up to 6 months after the surgery, patient recovered well and underwent regular oncologic outpatient clinic visits.

**Table 1:** Perioperative thyroid hormone levels

Item	Day of surgery	5th postoperative day	Reference range
FT3	0.32	0.65	1.80–4.10 pg/ml
FT4	0.17	0.22	0.81–1.89 ng/dl
T3	< 0.1	0.15	0.66–1.92 ng/ml
T4	1.00	1.40	4.30–12.50 ug/dl
TSH	92.592	> 150.000	0.380–4.340 uIU/ml
A-TPO	75		< 34 IU/ml
A-Tg	26		< 115 IU/ml

T3 = triiodothyronine, T4 = thyroxine, FT4 = free thyroxine, FT3 = free triiodothyronine, TSH = thyroid-stimulating hormone, A-TPO = anti-thyroid peroxidase antibodies, A-Tg = anti-Thyroglobulin Antibodies

## Discussion

This case underscores the significance of recognizing and addressing post-induction hypotension, which, in our case, could have been prevented or at least mitigated if we had known about the patient's immune-related adverse event (irAE)-related hypothyroidism due to Nivolumab, a PD-1 inhibitor.

In contemporary surgical settings, it's uncommon to encounter patients with severe hypothyroidism undergoing elective surgeries [8]. Hypothyroidism can lead to heightened sensitivity to anesthetic agents during general anesthesia [9], along with reduced medication metabolism, bradycardia, and difficulties in weaning off mechanical ventilatory support [10]. Most reported complications have occurred in patients with unrecognized hypothyroidism [11]. Refractory hypotension and cardiogenic shock due to hypothyroidism can be corrected by levothyroxine in a few days [10, 12]. Therefore preoperative recognition of hypothyroidism is essential for the safe anesthetic management of these patients.

Reflecting on this case, the bradycardia resulting from hypothyroidism also masked relative hypovolemia state due to Krukenberg tumor infraction. Hypothyroidism can blunt sympatho-excitatory and tachycardic responses to decreases in blood pressure [13]. Poor end vital organ perfusion resulting in elevated lactate level can also be found among with sever hypothyroidism [12], consistent with patient’s preoperative elevated lactate level though her vitals seems to be stable with BP ranging from 110 to 100 mmHg and HR ranging from 60 to 70 bpm. The blunted response to initial bolus of phenylephrine and ephedrine can also be supported by research evidence indicating diminished sensitivity to alpha-adrenergic agonist in hypothyroid patients [14].

This case also underscores the value of bedside ultrasound, which can help rule out hypovolemia and contractility dysfunction related to ischemic heart problems or valvular dysfunction, leading us to consider other uncommon cause of hypotension and pining the culprit of (irAE)-related hypothyroidism.

In the future for all the patients having history of taking PD-1, a quick preoperative work-up of irAE is need for emergency and elective surgeries alike and guidance on levothyroxine in dose, route of administration, and loading dose s needed. The current American Thyroid Association (ATA) guidelines recommend intravenous administration of levothyroxine at an initial dose of 200–400 µg. However, treatment should be pragmatic and adapted to the patient’s age, medical history, and critical illness condition.

## Conclusions

In emergent settings where time is limited to gather complete patient information, it is important to remain open-minded to the possibility of irAEs when treating patients receiving ICIs. This case also highlights the importance of POCUS in the initial diagnostic work-up of hepatic and renal complications, as in the present case. As the use of immune checkpoint inhibitors continues to expand, it is crucial for perioperative healthcare professionals to be aware of potential irAEs.

## Data Availability

Not applicable.

## Abbreviations

irAE: Immune-related adverse event
ICI: Immune checkpoint inhibitor
ECG: Electrocardiogram
T3: Triiodothyronine
T4: Thyroxine
FT4: Free thyroxine
FT3: Free triiodothyronine
TSH: Thyroid-stimulating hormone
A-TPO: Anti-thyroid peroxidase antibodies
A-Tg: Anti-Thyroglobulin Antibodies
TTE: Transthoracic echocardiogram
RWMA: Regional wall motion abnormality
ABG: Arterial blood gas
BP: Blood pressure
SPO2: Oxygen saturation
Hb: Hemoglobin
CK: Creatine kinase
POCUS: Point-of-care ultrasonography
